# Two New Clerodane Diterpenes from *Tinospora sagittata*

**DOI:** 10.3390/molecules21091250

**Published:** 2016-09-19

**Authors:** Guanhua Li, Wenbing Ding, Fanghao Wan, Youzhi Li

**Affiliations:** 1 College of Plant Protection, Hunan Agricultural University, Changsha 410128, China; liguanhua@hunau.edu.cn (G.L.); dingwenb119@hunau.edu.cn (W.D.); wanfanghao@caas.cn (F.W.); 2 Hunan Co-Innovation Center for Utilization of Botanical Functional Ingredients, Changsha 410128, China; 3 State Key Laboratory for Biology of Plant Diseases and Insect Pests, Institute of Plant Protection, Chinese Academy of Agricultural Sciences, Beijing 100193, China

**Keywords:** *Tinospora sagittata*, clerodane diterpenes, *α*-glucosidase inhibitory activity

## Abstract

Two new clerodane-type diterpenes, tinosporins C (**1**) and tinosporins D (**2**) were isolated from the stems of *Tinospora sagittata* (Oliv.), together with three known ones, columbin (**3**), tinophylloloside (**4**), and tinospinoside D (**5**). The structures of these compounds were determined on the basis of spectroscopic data interpretation, with that of the absolute configuration of compound **1** was assigned by experimental and calculated ECD spectra. The cytotoxicity and α-glucosidase inhibitory activities of isolated compounds were evaluated in vitro.

## 1. Introduction

*Tinospora sagittata* (Oliv.) Gagnep belonging to the family Menispermaceae, is a perennial indeciduous creeping vine widely distributed throughout the south of China and also found in northern Vietnam [1]. The roots of *T. sagittata* have been recorded in the traditional Chinese medicine known as “Jinguolan” which has the heat-clearing and detoxifying effects [2,3]. Plants of the genus *Tinospora* are known to be rich in clerodane diterpenes and the isolation of clerodane diterpenoids from *T. sagittata* has been reported in many different papers [4,5,6,7,8,9]. Nevertheless, different plant collection locations have yielded diterpenes with a variety of structures, and some of clerodane diterpenoid lactones also showed various bioactivities, such as anticancer [8,10], anti-inflammatory [5], and anti-adipogenic and anti-obesity activities [11]. As part of an ongoing search for novel bioactive compounds, we studied *T. sagittata* collected in Xiangxi, Hunan Province, China. Herein, this paper reports the isolation and identification of two *neo*-clerodane diterpenes **1** and **2** from the whole plant, as well as their cytotoxic and *α*-glucosidase inhibitory activities.

## 2. Results

**Figure 1:**
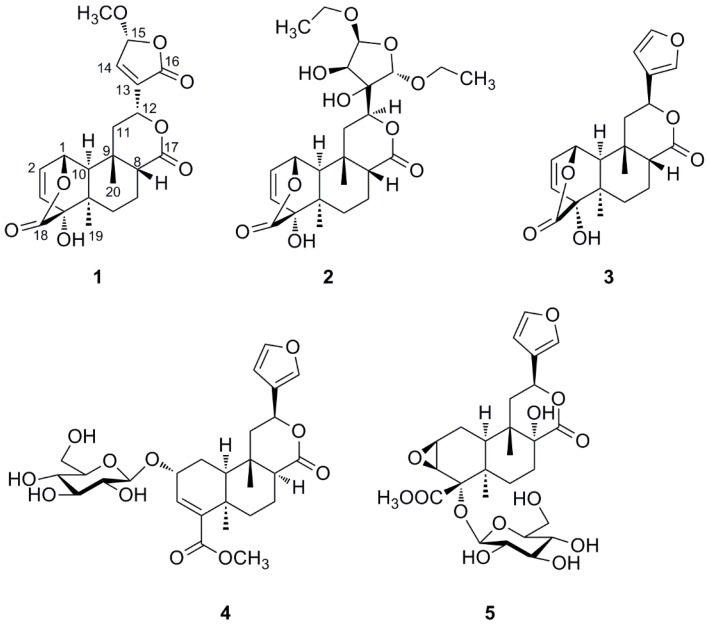
Structures of compounds **1**–**5** isolated from *T. sagittata*.

The dried whole plants of *T. sagittata* were extracted three times with ethanol at room temperature. The ethanol extract residue was suspended in water and then partitioned successively with petroleum ether, EtOAc, and *n*-BuOH. Column chromatography of the EtOAc-soluble fraction yielded two new clerodane diterpenoids **1**–**2** and three known ones **3**–**5** (Figure 1). The known compounds were identified as columbin (**3**) [12], tinophylloloside (**4**) [13], and tinospinoside D (**5**) [7] by comparing their physical and spectroscopic data with literature values.

**Figure 2:**
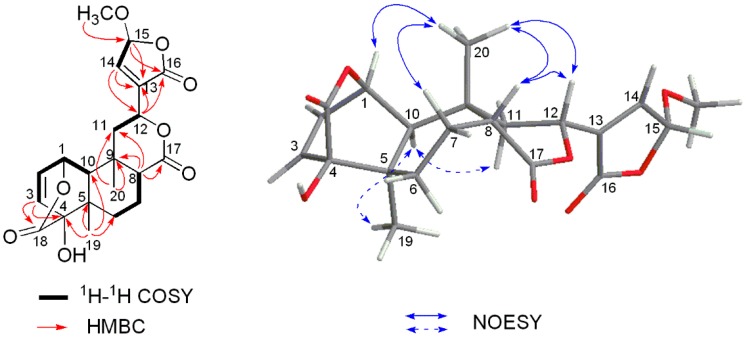
Key ^1^H-^1^H COSY, HMBC and NOESY correlations of **1**.

Compound **1** was obtained as colorless crystalline solids (CHCl_3_). The molecular formula was established as C_21_H_24_O_8_ based on the pseudo-molecular ion at *m*/*z* 427.1364 [M + Na]^+^ in the HR-ESIMS (calcd. for C_21_H_24_O_8_Na, 427.1363) (Figure S1), inferring ten degrees of unsaturation. The ^1^H-NMR spectrum of **1** (Figure S2) displayed recognizable signals for two angular methyl groups at δ_H_ 1.10 (3H, s) and 1.41 (3H, s), a methoxyl group at δ_H_ 3.58 (3H, s), and three olefinic proton signals at δ_H_ 6.34 (1H, overlapped), 6.33 (1H, overlapped) and 7.22 (1H, br s). The ^13^C-NMR and DEPT spectra of **1** (Figures S3 and S4) exhibited 21 carbons corresponding to three methyls, three carbonyl carbons, four olefinic carbons, three methylenes, five methines (including two oxy-methine carbons and a hemiacetal carbon), and three quaternary carbons (including an oxy-quaternary carbon) (Table 1). The aforementioned spectroscopic features revealed that compound **1** should be a clerodane-type furanoditerpene [14]. Comparison of the ^1^H- and ^13^C-NMR data with those of closely related analogues showed compound **1** had a basic structure closely similar to that of columbin, a major component of *T. sagittata* [12]. But the furan ring moiety attached C-12 in compound **1** was substituted by a 15-hydroxy-13(14)-ene-15,16-γ-lactone moiety which was proposed according to the resonances of an olefinic proton at δ_H_ 7.22 (1H, br s, H-14), 5.83 (1H, br s, H-15) in the ^1^H-NMR spectrum, as well as carbon signals of a double bond at δ_C_ 136.1 (C-13) and 144.7 (C-14), a carbonyl group at δ_C_ 168.6 (C-16), and a hemiacetal methane carbon at δ_C_ 103.2 (CH, C-15) in the ^13^C-NMR spectrum [15]. Moreover, an additional methoxyl moiety was found to be placed at C-15 which was established by the correlation between protons of -OC*H*_3_ (δ_H_ 3.58, 3H, s) and C-15 (δ_C_ 103.2) observed in the HMBC spectrum (Figure 2 and Figure S7).

The relative stereochemistry of **1** was assigned by analyses of the nuclear Overhauser effect spectroscopy (NOESY) correlation (Figure S8). The *cis* A/B ring junction was elucidated by the NOESY correlation between H-10 (δ_H_ 1.30) and H_3_-19 (δ_H_ 1.10), while the NOESY correlations of H-8/H_3_-20, H-8/H_3_-12, and H-12/H_3_-20, as well as the absence correlation between H-10 and H_3_-20 (δ_H_ 1.41) indicated the *cis* B/C ring fusion and the β-orientation of lactone moiety at C-12 (Figure 2).

**Figure 3:**
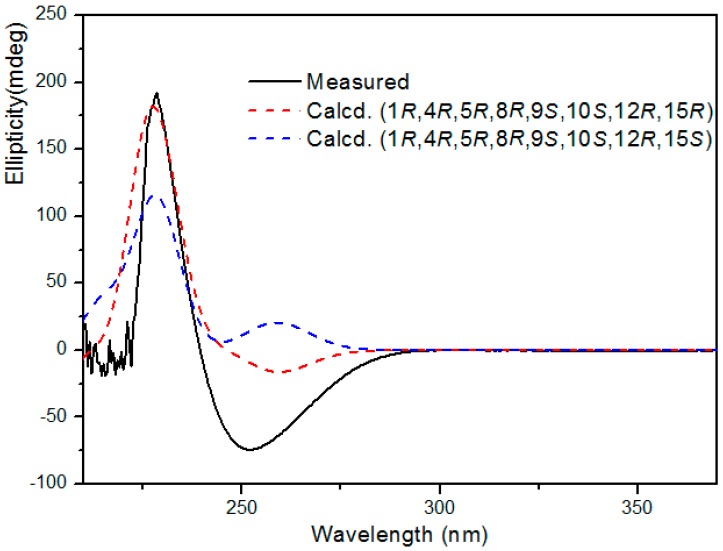
Experimental and calculated ECD spectra of compound **1**.

On the basis of the above relative configuration and biogenetic consideration, the absolute configuration of **1** was expected to be 1*R*,4*R*,5*R*,8*R*,9*S*,10*S*,12*R*,15*R* or 1*R*,4*R*,5*R*,8*R*,9*S*,10*S*,12*R*,15*S*. For the determination of the absolute configuration, the calculated ECD was performed using TDDFT performed with the Gaussian 09 program [16,17,18,19]. A comparison of the experimental ECD spectrum for **1** with the calculated ECD spectra for the corresponding 1*R*,4*R*,5*R*,8*R*,9*S*,10*S*,12*R*,15*R* and 1*R*,4*R*,5*R*,8*R*,9*S*,10*S*,12*R*,15*S* configurations (Figure 3), showed the calculated 1*R*,4*R*,5*R*,8*R*,9*S*,10*S*,12*R*,15*R* ECD spectrum matched well with the experimental one. Thus, the structure of compound **1** was unambiguously determined as shown in Figure 1 and it was named tinosporin C.

**Figure 4:**
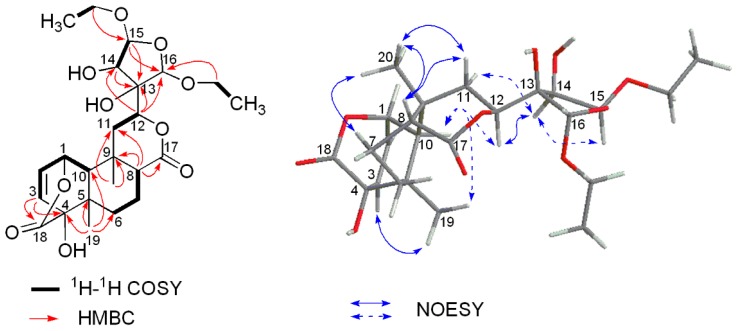
Key ^1^H-^1^H COSY, HMBC and NOESY correlations of **2**.

HR-ESIMS of compound **2** exhibited an [M + Na]^+^ ion peak at *m*/*z* 505.2044 (calcd. for 505.2044) (Figure S9), corresponding to a molecular formula of C_24_H_34_O_10_, which demonstrated eight degrees of unsaturation. The complete assignment of all the protons and carbons of **2** was accomplished by 2D NMR spectroscopy (including COSY, HSQC, and HMBC (Table 1)) which also revealed a basic structure of a *cis*-clerodane type diterpenoid, except that the furanoid ring moiety attached C-12 was replaced by a highly oxygenated tetrahydrofuran ring which was supported by crucial long-range correlations from H-12 to C-13, C-14 and C-16, from H-15 to C-13, C-14 and C-16 in the HMBC spectrum, together with correlation between H-14 (4.76 d, *J* = 4.2 Hz) and H-15 (5.59 d, *J* = 4.2 Hz) in ^1^H-^1^H COSY (Figure 4 and Figure S13). In addition, two additional ethoxy groups were established by the ^1^H-^1^H COSY spectrum based on the correlations of δ_H_ 3.54/3.91 (H_2_-methylene) with 1.17 (H_3_-methyl) and δ_H_ 3.57/3.89 (H_2_-methylene) with 1.14 (H_3_-methyl). The two ethoxy groups were deduced to be attached to C-15 and C-16 based on long-range correlations from the methylene protons (δ_H_ 3.54/3.91) to C-15 (δ_C_ 110.2) and from another methylene protons (δ_H_ 3.57/3.89) to C-16 (δ_C_ 106.9) observed in the HMBC spectrum (Figure S15), respectively. Key NOESY correlations (Figure 4 and Figure S16) of H-10/H_3_-19, H_3_-20/H-8, H_3_-20/Hβ-11 and H-8/Hβ-11, as well as absence of the NOESY correlations of H_3_-20/H-10, suggested the relative configuration of the A, B, and C rings in **2** remained the same as those in columbin [12]. The NOESY correlation of H-10/H-12 suggested the β-equatorial orientation of the highly oxygenated tetrahydrofuran at C-12. The relative stereochemistry of the tetrahydrofuran moiety was further deduced based on correlations of H-12/H-14, H-14/H-15, and the absence of correlations of H-14/H-16 and H-14/H-16 observed in the NOESY spectrum. Finally, the structure of compound **2** was determined to be as shown in Figure 1 and named tinosporin D.

The isolated compounds **1**−**5** were tested for their α-glucosidase inhibitory activity. Compounds **1** and **5** showed inhibition effects by 48.58%, 47.08% at a concentration of 20 µg/mL, respectively, which is weaker than that of the positive control acarbose (79.09% at 20 µg/mL). Compounds **1** and **2** were further tested for their cytotoxicity against three human tumors HepG2 (human liver hepatocellular carcinoma), HCT116 (human colon carcinoma) and SGC-7901 (human gastric carcinoma) cell lines, using a sulforhodamine B (SRB) method. The results indicated that both were inactive at 100 μM.

## 3. Materials and Methods

### 3.1. General Information

Optical rotations were determined using a Perkin-Elmer 341 polarimeter (PerkinElmer Co., Waltham, MA, USA). Melting points were taken on a SGW X-4 micromelting point apparatus (INESA Physico Optical Instrument Co., Ltd., Shanghai, China) and HRESIMS spectra on an API QSTAR mass spectrometer (Applied Biosystem/MSD Sciex, Concord, ON, Canada). ECD spectra were recorded at 25 °C on a MOS-450/SFM300 spectrophotometer (Bio-logic, Claix, France). The ^1^H, ^13^C, and 2D NMR spectra were recorded on a Bruker DRX-400 instrument using TMS as an internal standard. Column chromatography was performed on silica gel 60 (200–300 mesh, Qingdao Marine Chemical Ltd., Qingdao, China). Preparative HPLC was performed on a Waters 1525 Binary HPLC pump and a Waters 2414 refractive index detector using a YMC-Pack ODS-A column (250 mm × 10 mm I.D.; S-5 μm, 12 nm). For Preparative TLC plates (HSGF254, Jiangyou silicone Development Co., Ltd., Yantai, China), Sephadex LH-20 (GE Healthcare, Uppsala, Sweden) and Develosil ODS (50 μm, Nomura Chemical Co. Ltd., Osaka, Japan) were used. 

### 3.2. Plant Material

The whole plants of *T. sagittata* were collected from Xiangxi, Hunan Province, China, in August 2012, and identified by Prof. Dai-gui Zhang (Key Laboratory of Plant Resources Conservation and Utilization, Jishou University, Jishou, China). A voucher specimen (zdg201208-02) has been deposited in Hunan Agricultural University.

### 3.3. Extraction and Isolation

The fresh whole plant of *T. sagittata* (4.8 kg) was dried at 40 °C. The dried materials were powdered and extracted three times with EtOH (95% *v*/*v*) at room temperature, then the combined extract was evaporated under reduced pressure to obtain a crude residue (347.4 g). The residue was further suspended in H_2_O (2 L) and then successively extracted with petroleum ether (PE), EtOAc and *n*-BuOH to yield PE-soluble fraction 93.0 g, EtOAc-soluble fraction 54.7 g and *n*-BuOH-soluble fraction 60.8 g, respectively. The EtOAc-soluble fraction was subjected to silica gel column chromatography (CC) (100–200 mesh) with elution of CHCl_3_–MeOH (100:0→60:40, *v*/*v*) to give seven fractions (Fr. A–G). Faction B (4.5 g) was further separated by repeated silica gel CC (CHCl_3_–MeOH, 10:0→9:1 gradient system, 200–300 mesh), and then compound **3** (150 mg) was recrystallized from subfraction B-2. Fraction C (3.5 g) was fractionated by an ODS-C_18_ column with elution of MeOH–H_2_O (30:70→70:30, *v*/*v*) to give five subfractions Fr. C-1 to C-5. Subfraction C-2 was performed on Sephadex LH-20 column with elution of MeOH to obtain **1** (19 mg). Similarly, compound **2** (51 mg) was obtained from subfraction C-3 which was purified successively on Sephadex LH-20 column (MeOH) and repeated silica gel CC (CHCl_3_–MeOH, 10:0→9:1, 200–300 mesh). Fraction E was subjected to ODS-C_18_ CC (40% MeOH in H_2_O) to give three subfractions Fr. E-1 to E-3. Subfraction E-2 was subsequently purified by Sephadex LH-20 column (MeOH) and semi-preparative HPLC (MeOH–H_2_O, 35:65, *v*/*v*, flow rate 3 mL/min) to afford **5** (19 mg, *t*_R_ = 24 min) and **4** (79 mg, *t*_R_ = 28 min). In order to confirm that the two new compounds (**1**–**2**) are original natural products, a re-examination of **1** and **2** in the plant materials was performed without using of methanol or ethanol [16].

### 3.4. Spectroscopic Data of ***1*** and ***2***

Compound **1**: colorless crystalline solid, mp 182–185 °C, [α]${}_{D}^{25}$ +26.7 (*c* 0.15, MeOH); ECD (MeOH) λ (Δε) 228.5 (+192.2), 252 (−74.2); ^1^H-NMR (400 MHz, CDCl_3_) and ^13^C-NMR (100 MHz, CDCl_3_) spectroscopic data see Table 1; positive ion ESIMS *m*/*z*: 427 [M + Na]^+^ and 831 [M + Na]^+^; HR-ESIMS *m*/*z*: 427.1364 [M + Na]^+^ (calcd for C_21_H_24_O_8_Na 427.1363). 

Compound **2**: Colorless crystals, mp 197–198 °C, [α]${}_{D}^{25}$ +31.2 (*c* 0.16, MeOH); ^1^H-NMR (400 MHz, C_5_D_5_N) and ^13^C-NMR (100 MHz, C_5_D_5_N) spectroscopic data see Table 1; positive ion ESIMS *m*/*z*: 505 [M + Na]^+^; negative ESIMS *m*/*z*: 481 [M − H]^−^, 963 [2M + Cl]^−^; HR-ESIMS *m*/*z*: 505.2044 [M + Na]^+^ (calcd for C_24_H_34_O_10_Na, 505.2044).

### 3.5. Computational methods for ECD of Compound ***1***

The CONFLEX [17,18] searches based on molecular mechanics with MMFF94S force fields were performed for **1**, which gave 8 stable conformers. Selected conformers of 1 with the lowest energy were further optimized by the density functional theory method at the B3LYP/6-31+G (d, p) level in Gaussian 09 program package [19], which was further checked by frequency calculation and resulted in no imaginary frequencies. The ECD of the conformer of **1** was then calculated by the TDDFT method at the B3LYP/6-31G (d, p) level with the CPCM model in methanol solution. The calculated ECD curve was generated using SpecDis 1.51 [20] with σ = 0.20 eV.

### 3.6. α-Glucosidase Inhibition Assay

The α-glucosidase inhibitory activity of **1**−**5** was determined following the method described in the literature with slight modifications [21]. In brief, α-glucosidase (50 μL, 0.5 U/mL) and a concentration 100 μg/mL of tested compounds (50 μL) in phosphate buffer (pH 6.8) were mixed at room temperature for 10 min. Reactions were initiated by addition of 5.0 mM *p*-nitrophenyl-α-d-glucopyranoside (PNPG, 150 μL). The reaction mixture was incubated for 15 min at 37 °C in a final volume of 250 μL. Then, 0.2 M Na_2_CO_3_ (100 μL) was added to the incubation solution to stop the reaction. The activities were detected in a 96-well plate, and the absorbance was determined at 405 nm (for *p*-nitrophenol). The negative blank was set by adding phosphate buffer instead of the sample via the same way as the test. Acarbose was utilized as positive control. The blank was set by adding phosphate buffer instead of the α-glucosidase using the same method. Inhibition rate (%) = [(OD_negative control_ − OD_blank_) − (OD_test_ − OD_test blank_)]/(OD_negative blank_ − OD_blank_) × 100%.

### 3.7. Cytotoxicity Assay

The cytotoxicity of the new compounds **1** and **2** against three human cancer cell lines, human gastric carcinoma (SGC-7901), human colon carcinoma (HCT 116) and human liver hepatocellular carcinoma (HepG2) were assayed at the National Center for Drug Screening, Shanghai, China. Sulforhodamine B (SRB) (Sigma-Aldrich Chemie GmbH, Munich, Germany) was used to test the effects of the compounds on cell growth and viability [22]. Adriamycin was used as the positive control.

## 4. Conclusions

The phytochemical investigation of whole plant of *T. sagittata* afforded two new clerodane-type diterpenes, tinosporin C (**1**) and tinosporin D (**2**), as well as three known ones, columbin (**3**), tinophylloloside (**4**), and tinospinoside D (**5**). Bioassays showed that compounds **1** and **5** display slight in vitro α-glucosidase inhibitory activities. The discovery of the new compounds expands our knowledge of the structural diversity of the clerodane-type diterpenes produced by the plant *T. sagittata*.

## Figures and Tables

**Table 1:** ^1^H- (400 MHz) and ^13^C- (100 MHz) NMR spectral data of compounds **1** and **2**.

No.	1 ^a^		No.	2 ^b^	
	δ_H_	δ_C_		δ_H_	δ_C_
1	5.10 br t, 3.4	74.8	1	5.30 dd, *J* = 5.2, 1.7	73.2
2	6.34 overlapped	128.3	2	6.23 dd, *J* = 7.9, 5.2	129.3
3	6.33 overlapped	137.3	3	6.42 dd, *J* = 7.9, 1.7	136.9
4		80.7	4		80.8
5		37.8	5		37.1
6 (β-H)	1.35 m	26.0	6 (β-H)	1.79 m	26.0
6 (α-H)	1.76 ddd, *J* = 14.2, 6.6, 2.0		6 (α-H)	2.03 m	
7 (α-H)	1.94 m	17.1	7 (α-H)	2.01 m	17.5
7 (β-H)	2.53 m		7 (β-H)	2.85 m	
8	2.55 m	43.6	8	2.52 m	44.1
9		36.3	9		34.1
10	1.30 br s	55.3	10	2.00 br s	46.8
11 (β-H)	2.48 dd, *J* = 14.0, 2.6	45.6	11 (α-H)	2.60 dd, *J* = 14.8, 5.6	34.0
11 (α-H)	1.64 m		11 (β-H)	2.54 m	
12	5.22 br d, *J* = 11.5	70.2	12	5.42 dd, *J* = 14.8, 5.6	76.8
13		136.1	13		80.6
14	7.22 br s	144.7	14	4.76 d, *J* = 4.2	76.4
15	5.83 br s	103.2	15	5.59 d, *J* = 4.2	110.2
16		168.6	16	5.57, s	106.9
17		173.7	17		173.7
18		175.6	18		174.8
19	1.10 s	24.1	19	1.23 s	23.9
20	1.41 s	28.3	20	1.22 s	27.2
15-OCH_3_	3.58 s	57.4	15-*O***CH_2_**CH_3_	3.54 m; 3.91 m	63.7
			15-*O*CH_2_**CH_3_**	1.17 t, *J* = 7.1	14.7
			16-*O***CH_2_**CH_3_	3.57 m; 3.89 m	62.4
			16-*O*CH_2_**CH_3_**	1.14 t, *J* = 7.1	14.4

^a^ measured in CDCl_3_, ^b^ measured in C_5_D_5_N; δ in ppm and *J* in Hz.

## Supplementary Materials

The HR-ESIMS, ^1^H- and ^13^C-NMR and 2D-NMR spectra (Figures S1–S16) of compounds **1** and **2** are available at http://www.mdpi.com/1420-3049/21/9/1250/s1.
