# Supplementary material for: Two New Clerodane Diterpenes from Tinospora sagittata
Source: Molecules. 2016 Sep 19;21(9):1250. doi: 10.3390/molecules21091250 (PMC6274121; doi:10.3390/molecules21091250)
Supplement: Supplementary file 1 [file molecules-21-01250-s001.pdf]

Supplementary Materials: Two New Clerodane Diterpenes from *Tinospora sagittata*

Guanhua Li, Wenbing Ding, Fanghao Wan and Youzhi Li

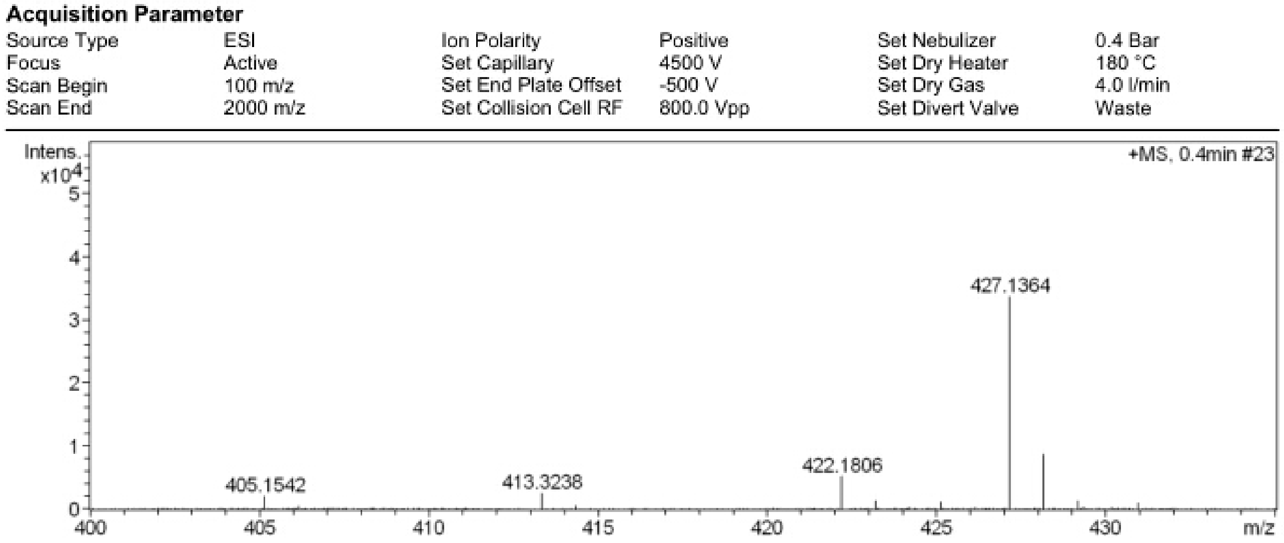

Figure S1. HR-ESIMS of compound **1**.

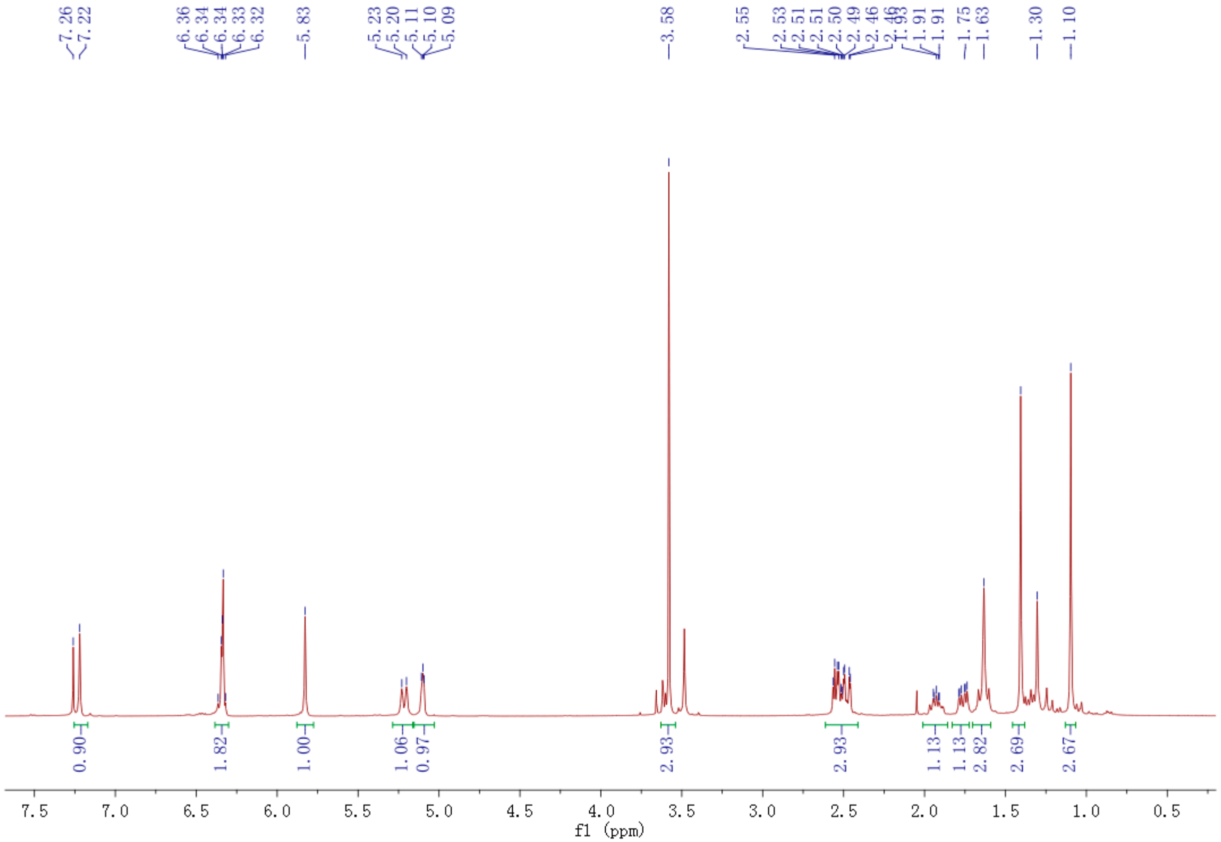

Figure S2. ^{1}H-NMR of compound **1**.

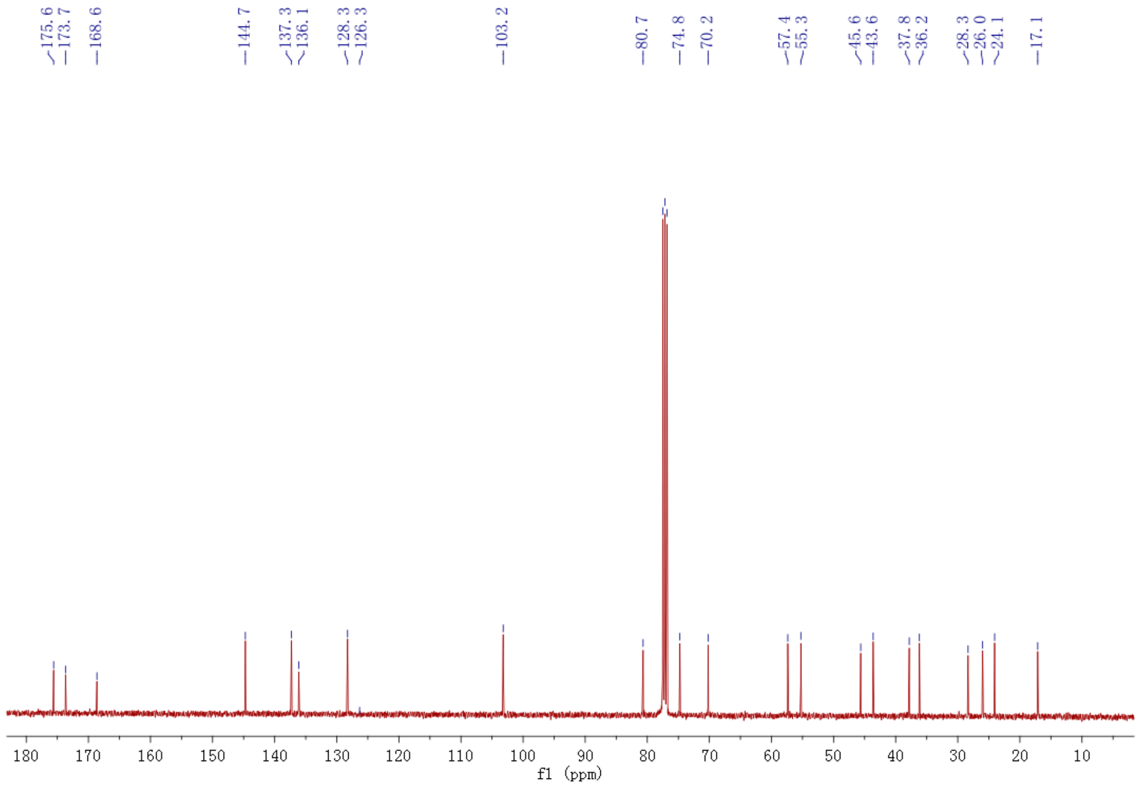

Figure S3. ^{13}C-NMR of compound **1**.

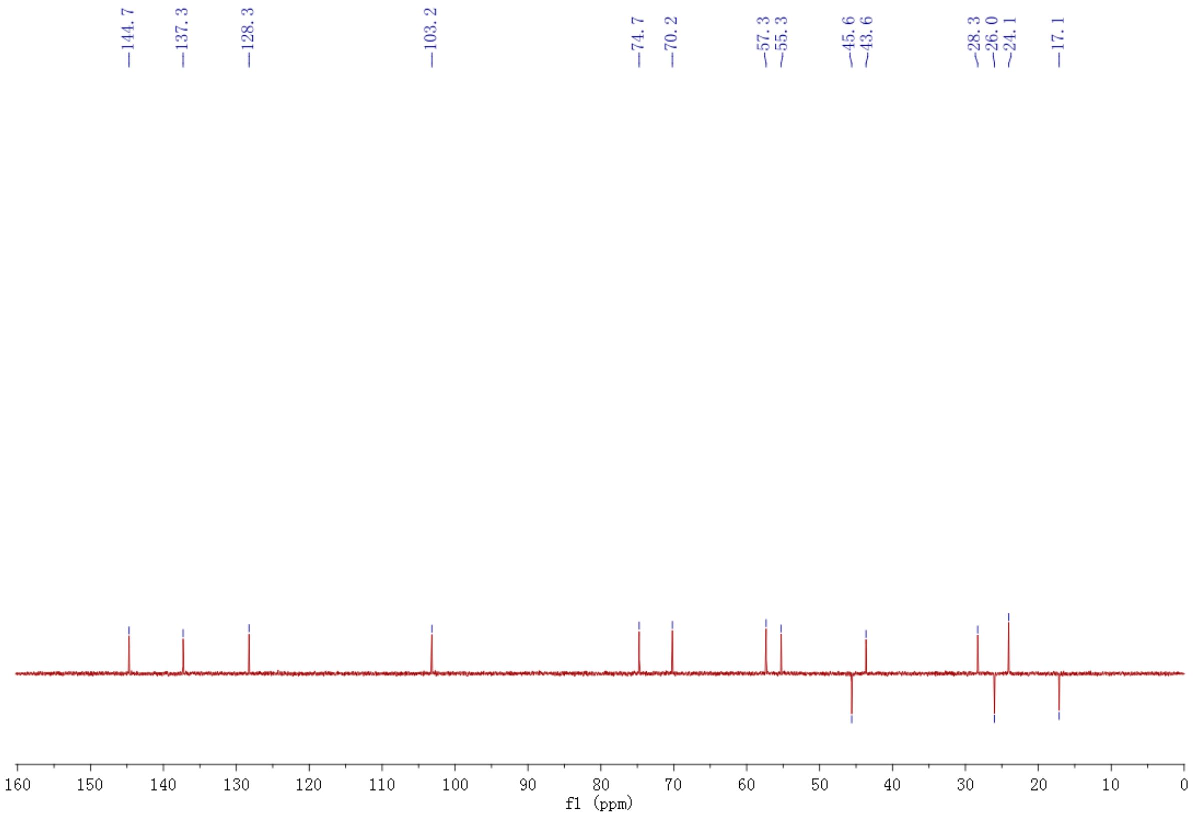

Figure S4. DEPT-135 of compound **1**.

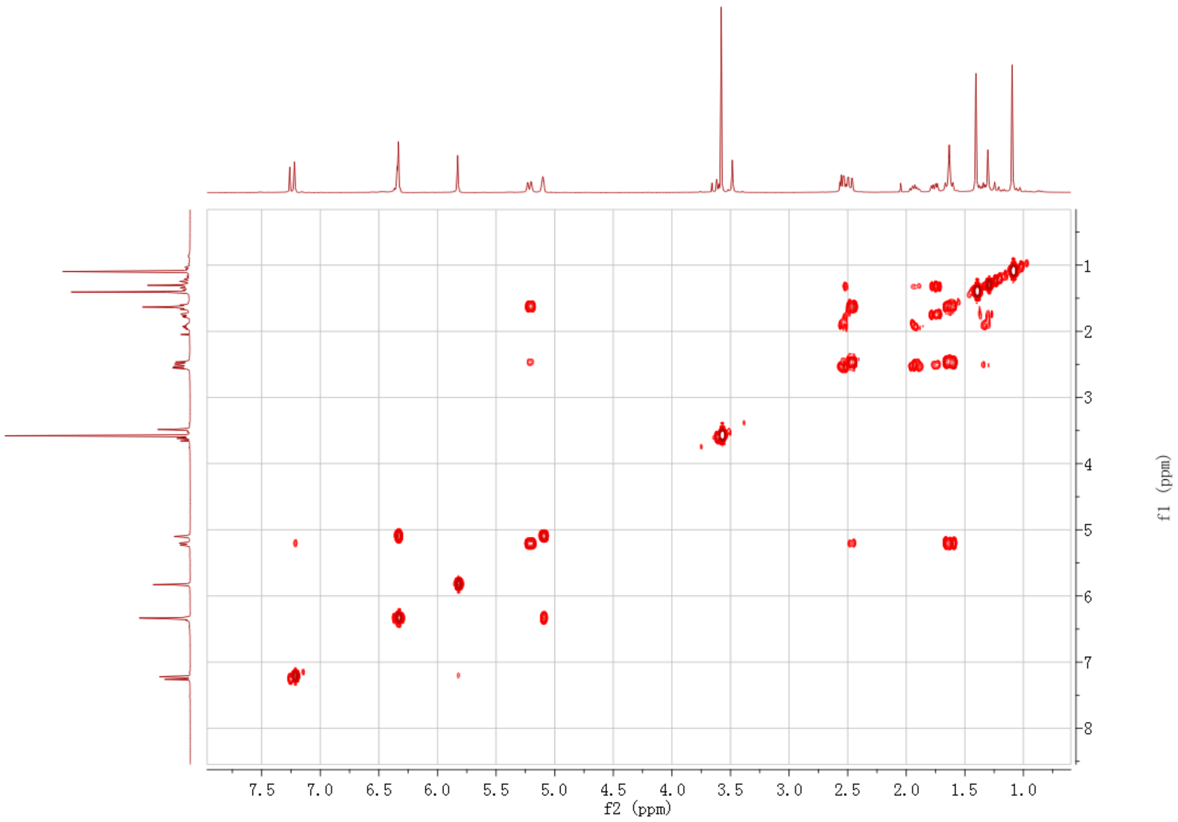

Figure S5. ^{1}H-^{1}H COSY of compound **1**.

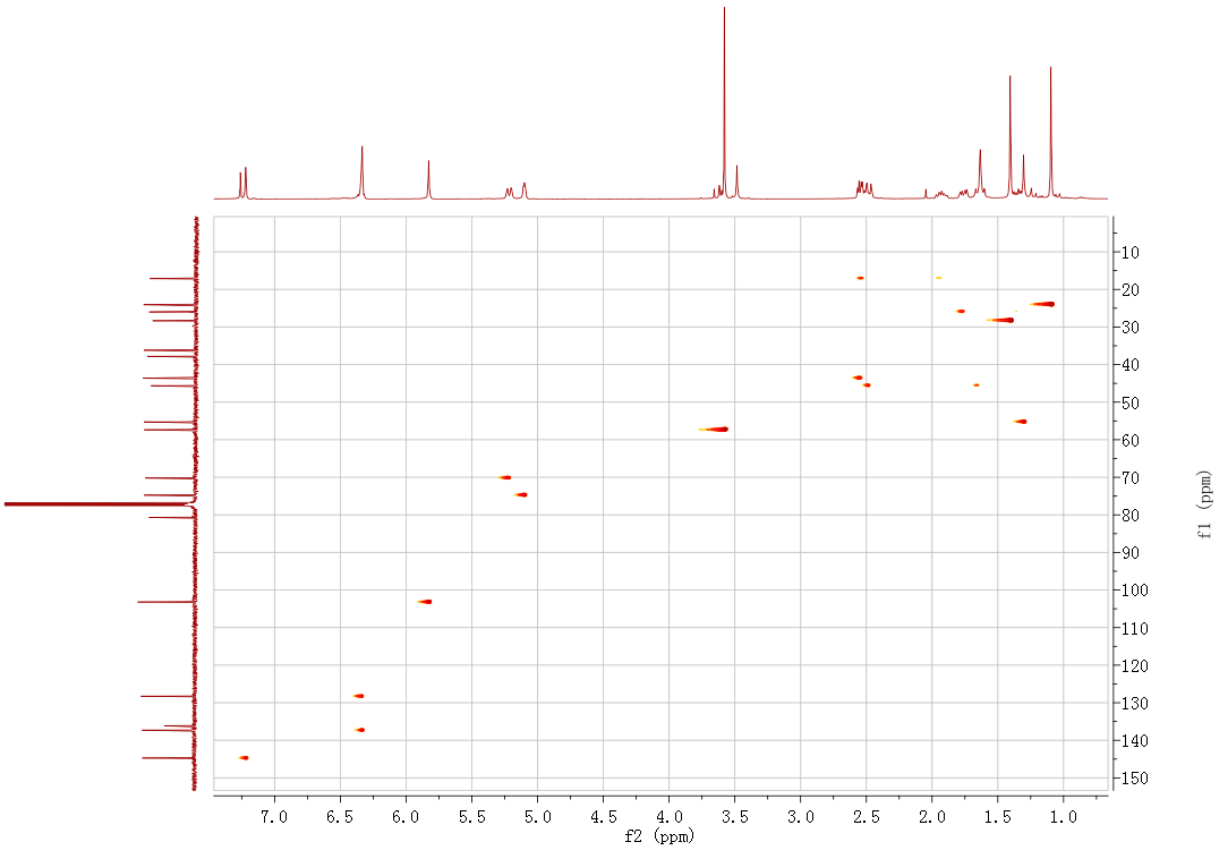

Figure S6. HSQC of compound **1**.

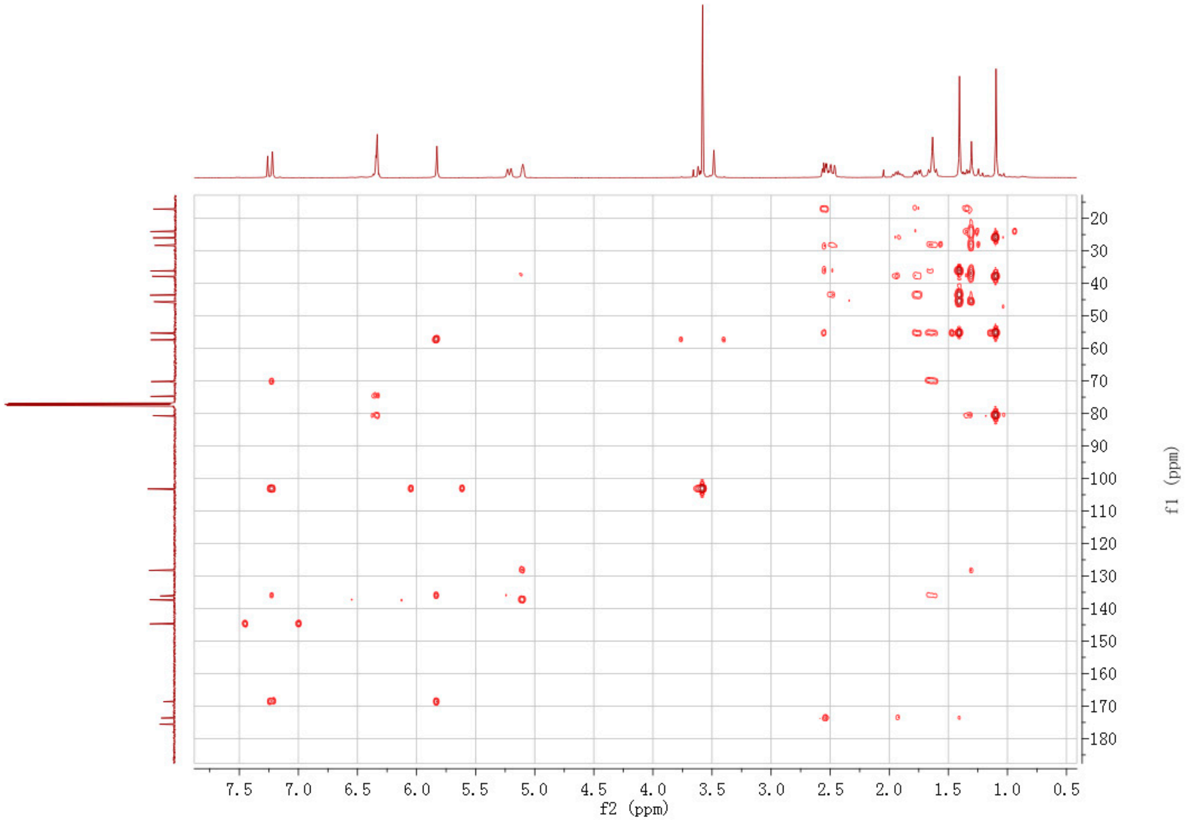

Figure S7. HMBC of compound **1**.

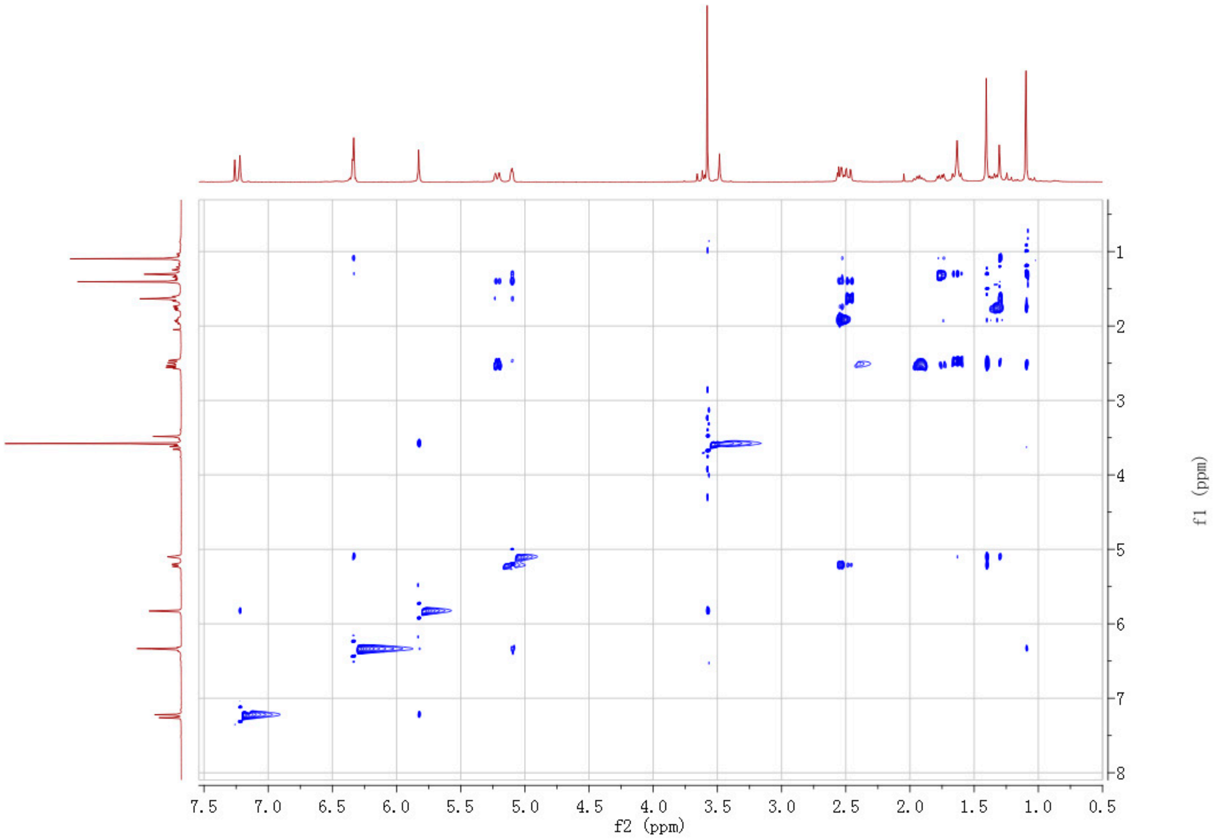

Figure S8. NOESY of compound **1**.

Acquisition Parameter

Source Type	ESI	Ion Polarity	Positive	Set Nebulizer	0.4 Bar
Focus	Active	Set Capillary	4500 V	Set Dry Heater	180 °C
Scan Begin	100 m/z	Set End Plate Offset	-500 V	Set Dry Gas	4.0 l/min
Scan End	2000 m/z	Set Collision Cell RF	800.0 Vpp	Set Divert Valve	Waste

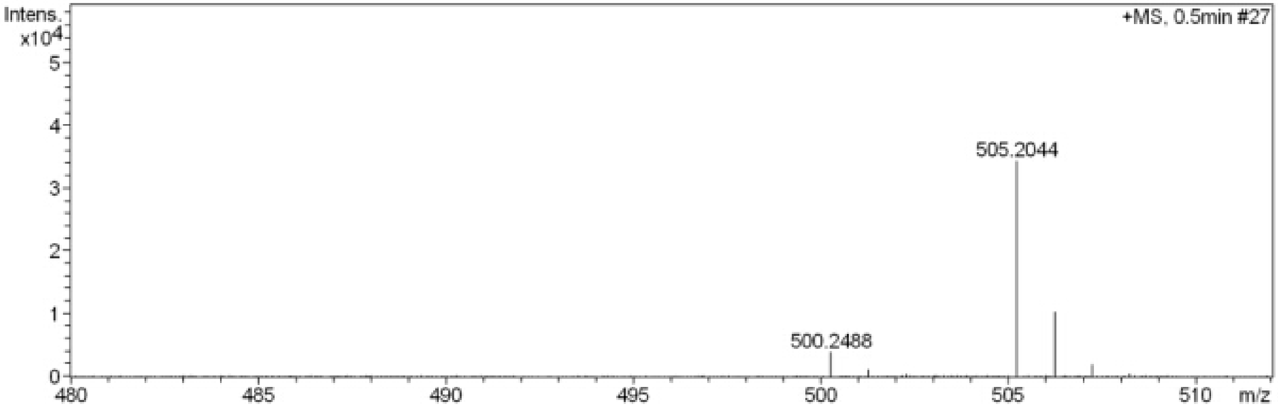

Figure S9. HR-ESIMS of compound **2**.

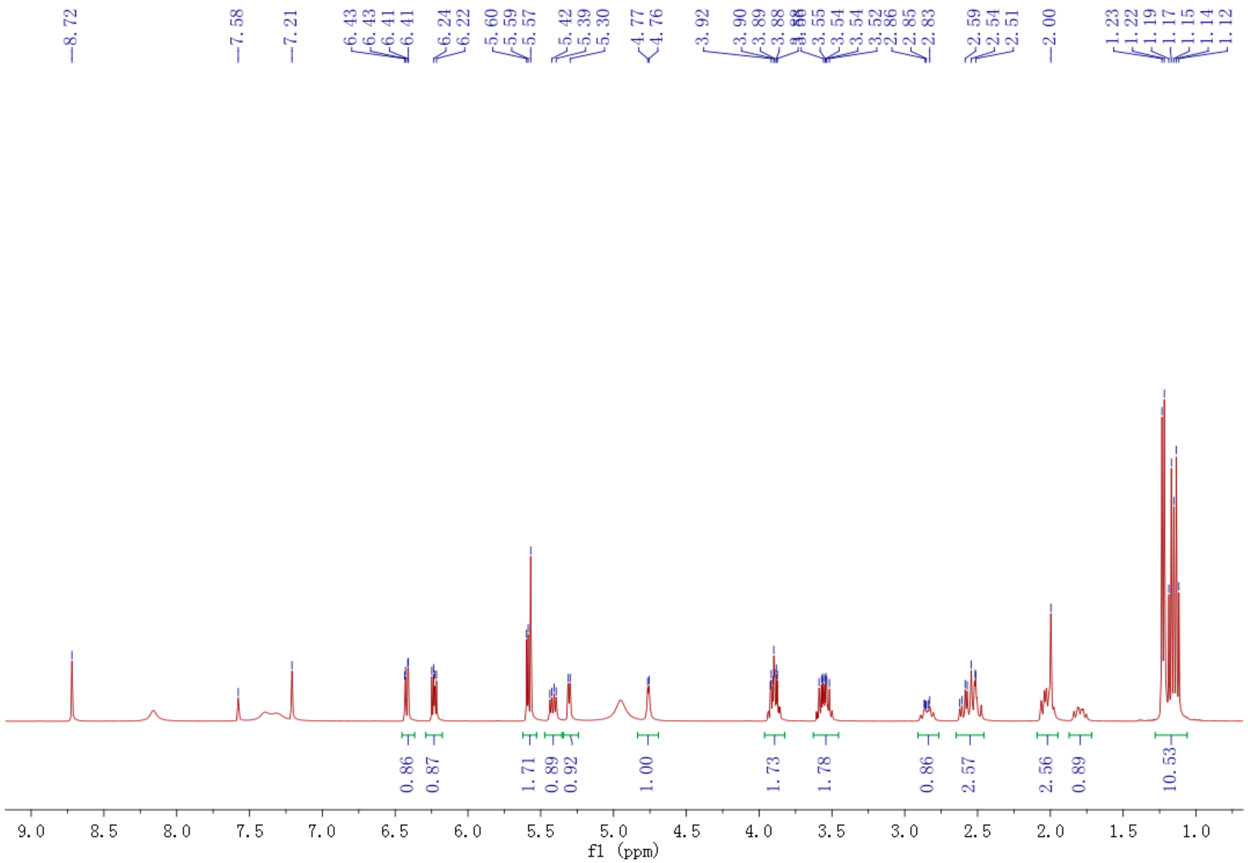

Figure S10. ^{1}H-NMR of compound **2**.

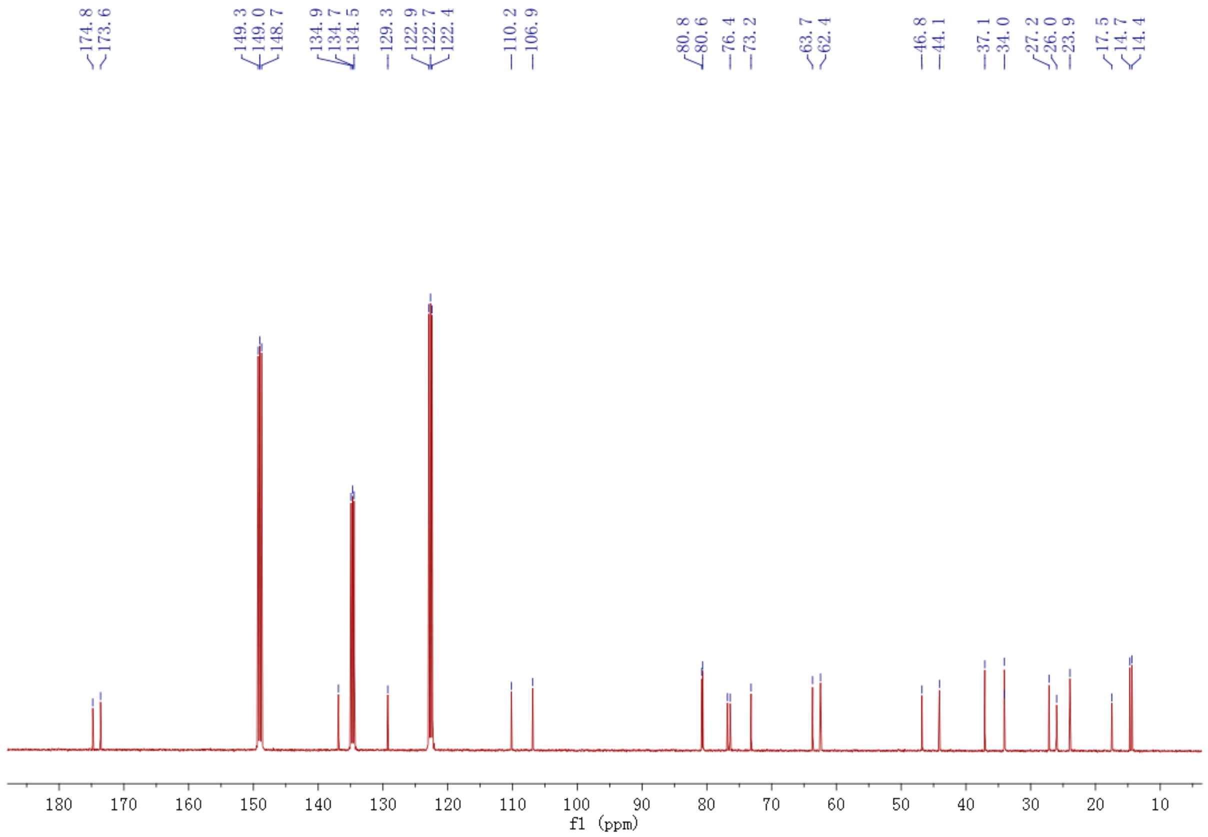

Figure S11. ^{13}C-NMR of compound **2**.

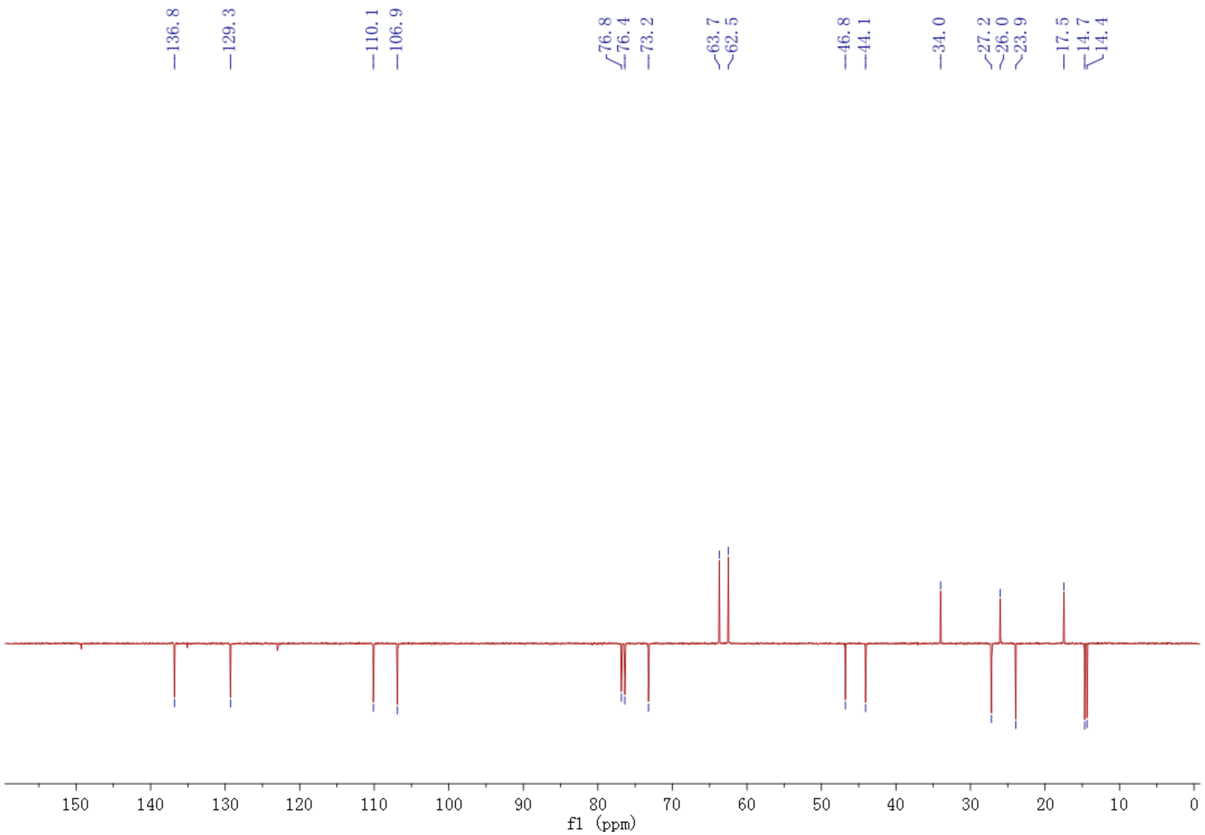

Figure S12. DEPT-135 of compound **2**.

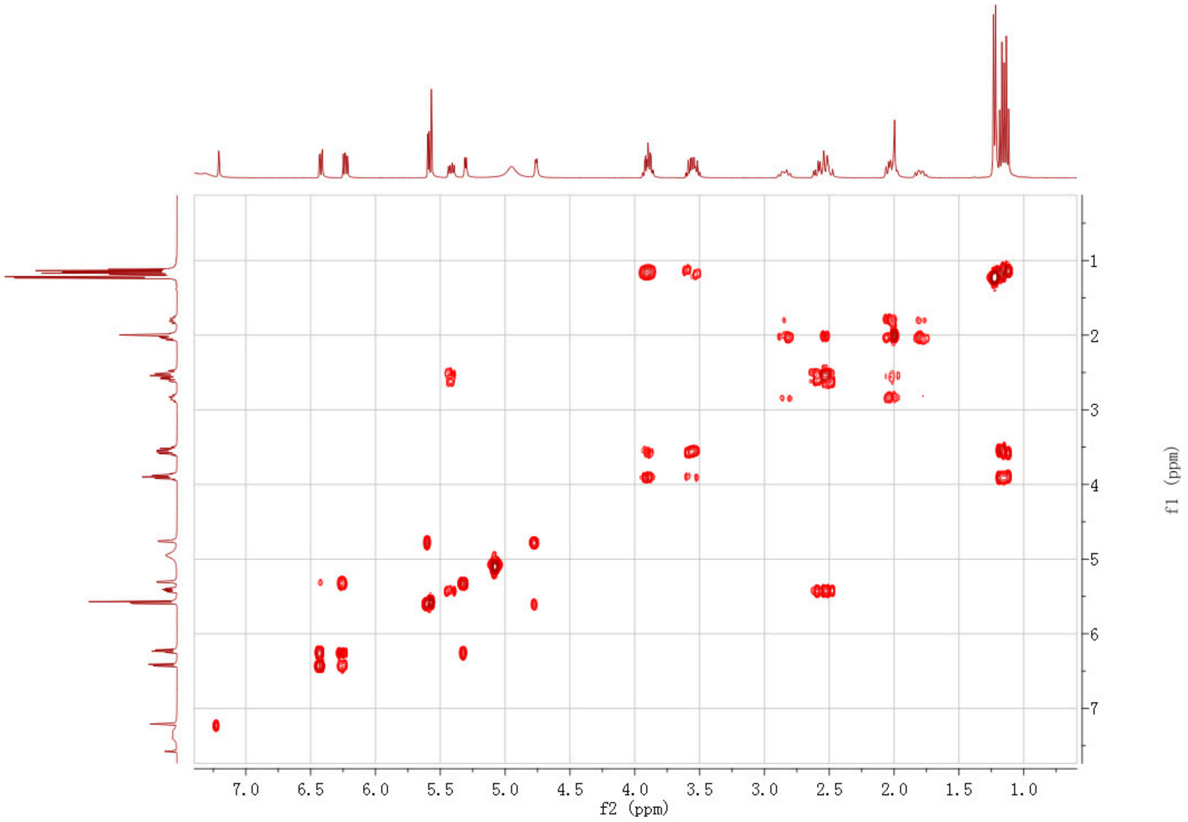

Figure S13. ^{1}H-^{1}H COSY of compound **2**.

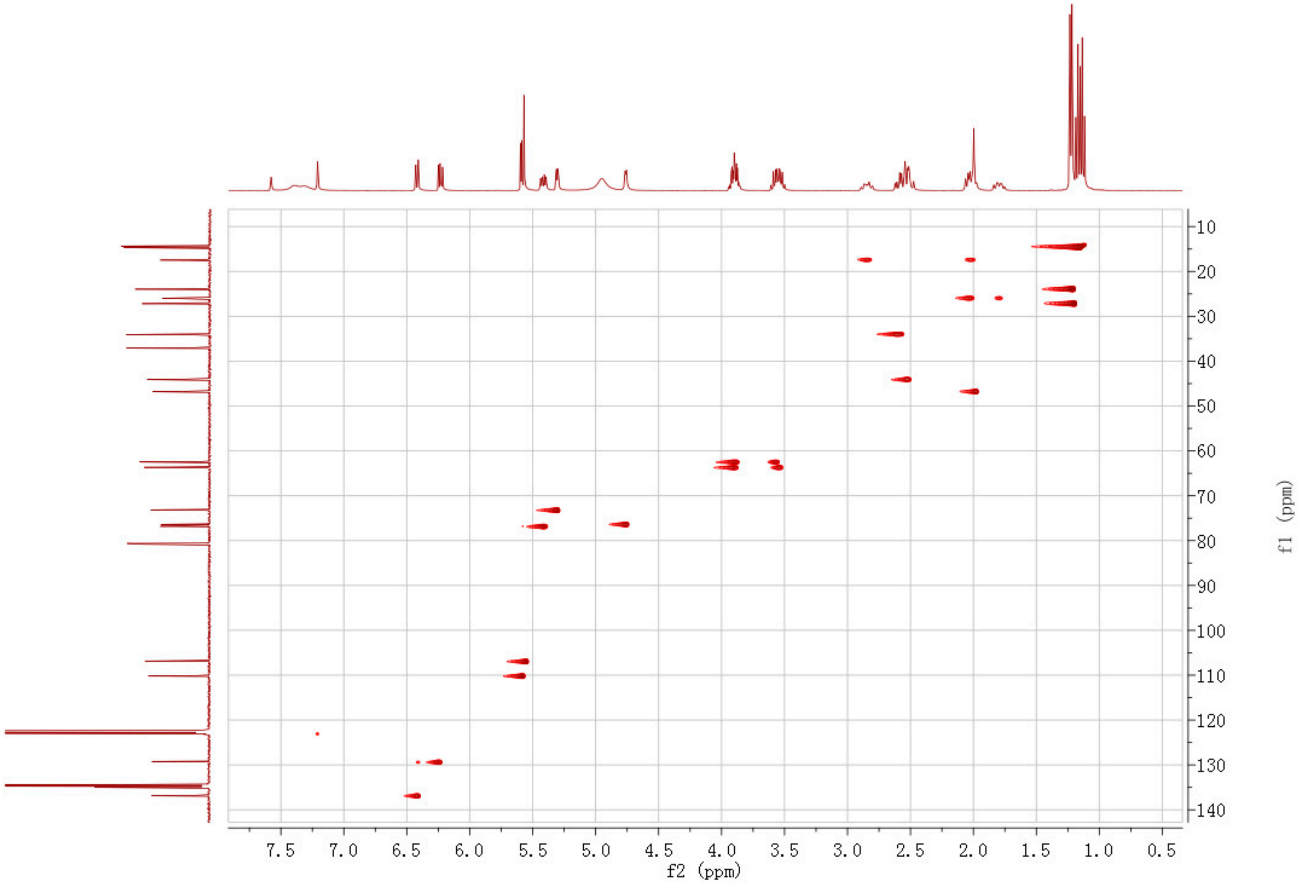

Figure S14. HSQC of compound **2**.

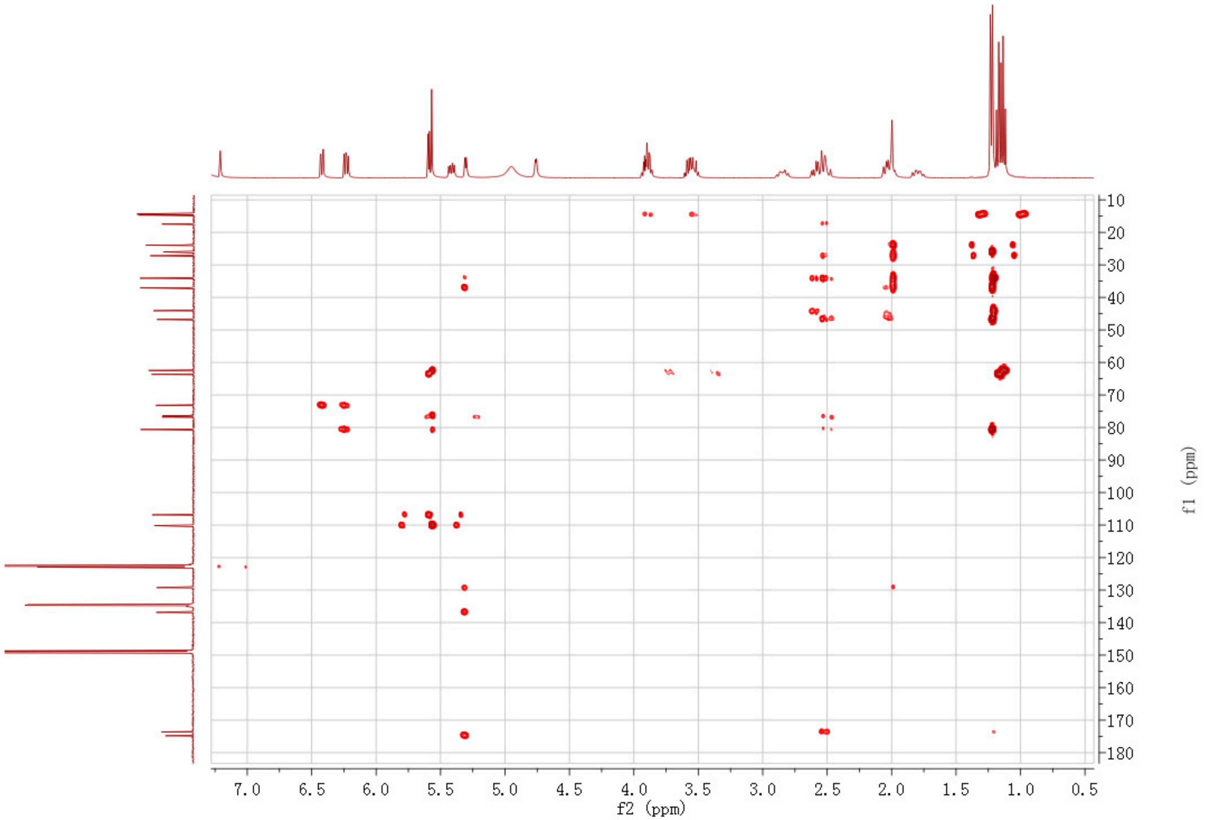

Figure S15. HMBC of compound **2**.

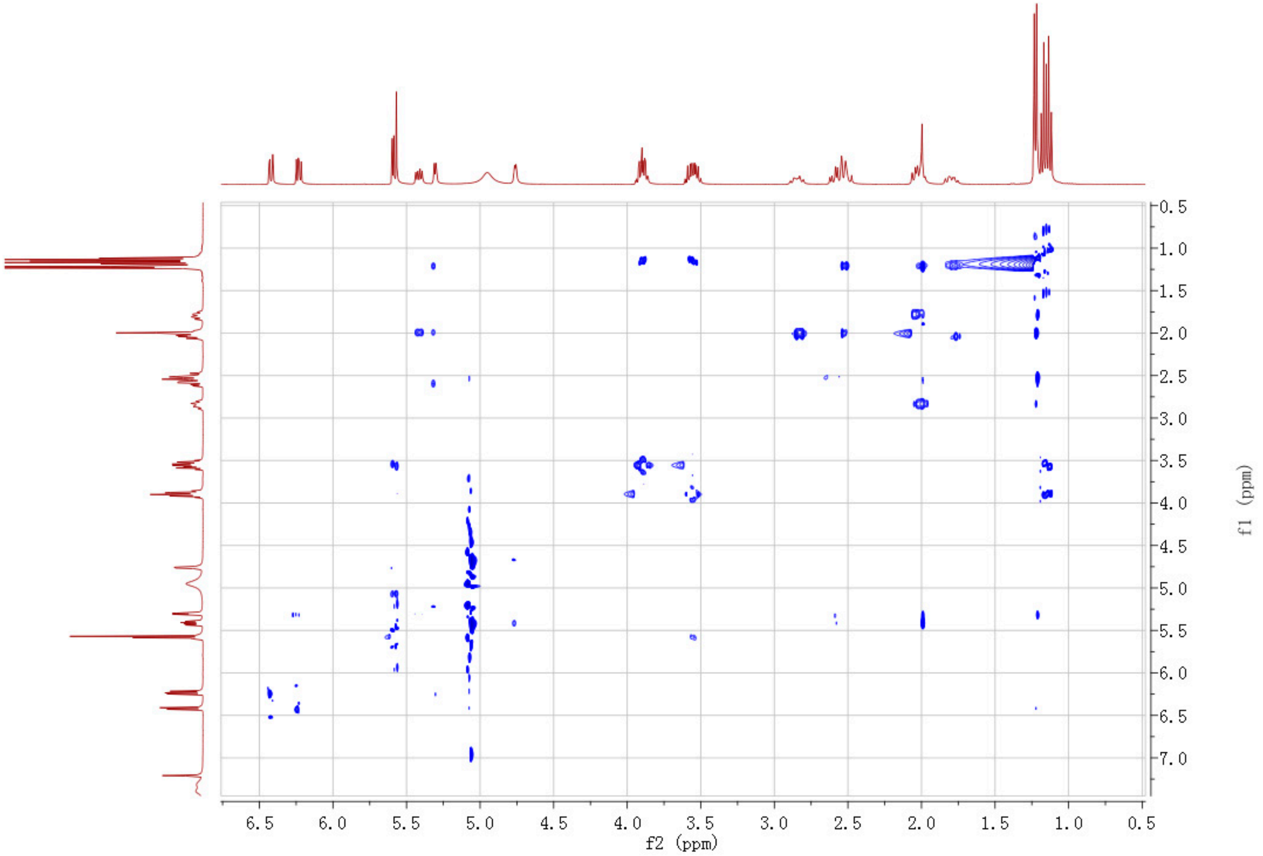

Figure S16. NOESY of compound **2**.
